# Webster’s Unabridged Dictionary

**Published:** 1868-10

**Authors:** 


### WEBSTER'S UNABRIDGED DICTIONARY.

3000 ENGRAVINGS. 1840 pp. QUARTO. TRADE PRICE $12.

THE NEW WEBSTER is glorious—it is perfect—it distances and defies competition—it leaves nothing to be desired—*F. H. Raymond, LL.D., President Vassar College.*

COMPENDIUM OF HUMAN KNOWLEDGE.—Webster's Unabridged Dictionary must be regarded as the most useful and remarkable *Compendium of human knowledge* in our language. —*W. S. Clark Pres. Mass. Agricultural College.*

THE NEW ILLUSTRATED EDITION OF WEBSTER, *a library for the poor man*, and an indispensable piece of furniture for the table of the scholar.—*Geo. Woods, LL. D., Pres'. Western University, Pa.*

It is the Dictionary of dictionaries: *I have fallen in love with it.* So has my wife, and so has my children.—*F. C. Packard, late Sup't. Public Instruction, Wisconsin.*

Viewed as a whole, we are confident that no other living language has a dictionary which so fully and faithfully sets forth its present condition as this last edition of Webster does that of our written and spoken English tongue.—*Harper's Magazine.*

BEST BOOK FOR EVERYBODY.—The new illustrated edition of Webster's Dictionary, containing three thousand engravings, is the *best book for everybody* that the press has produced in the present century.—*Golden Era.*

Young man, if you already have a Bible, buy Webster's Unabridged Dictionary next.—*Chr. Sun.*

Published by G. & C. MERRIAM, Springfield Mass. Sold by all booksellers. Also, just published, WEBSTER'S NATIONAL PICTORIAL DICTIONARY 1040 pp.; Octavo; 600 Engravings. Trade Price $6.

*The Presbyterian Historical Almanac for* 1867, vol. nine, by Joseph M. Wilson, No. 123 South Fourth St. Philadelphia, Pa., baing an annual remembrancer of the Church, is a publication of standard value, and becomes of greater interest with each passing year, as it catches up a great variety of historical matter and preserves for future reference what otherwise would have been lost or in various ways would become useless for all practical purposes. For example, a thousand ministers have died since the publication of the first volume, of each of whom something has been said, ranging from a brief notice to a comprehensive biography ; and who can tell with what intense interest these will be read by the descendants of the honored names, it may be for centuries to come. Then there are portraits of many clergymen who have lived and toiled and died in the master's service, and whose works do follow, besides the "minutes" of many church judicatories, assemblies, synods, conferences &c. The vol. for 1867 contains 531 pages 8 vo. of reading matter and merits a place in the library of the clergyman, the elder and the educated christian gentleman, and we feel sure that Mr. Wilson merits the respect of the whole church for this his labor of love conducted from year to year at a pecuniary loss.

We copy the following notice of our leading retail clothier from the *Hempstead Inquirer*, (L. I.) We commend the rules which govern Mr. Baldwin's house to merchants generally.

WE LIKE THE PRINCIPLES.

We had heard so much within the past few months concerning the famous corner, Broadway and Canal Street, we called there a few days since and had a few minutes chat with the proprietor. We asked Mr. Baldwin the secret of his success. "In the first place," said he, "I intend to have the *lowest* prices in New York, or elsewhere, for first-class clothing, and there shall be *one price* only. I pay *no commissions on sales* to any one. No misrepresentations are allowed under any circumstances in selling the goods, and we make no distinction among customers—*all are treated politely*. Our terms are 'CASH ON DELIVERY,' thereby keeping us upon friendly terms with *all* our patrons. And," added Mr. Baldwin, "I am determined to *keep down* prices, and make *this corner* the most attractive place in the city for ready-made, stylish, and cheap clothing." We commend our readers to BALDWIN THE CLOTHIER, *corner Broadway and Canal Street.*

